# Prognostic significance of preoperative neutrophil-to-lymphocyte ratio and platelet-to-lymphocyte ratio in patients with glioma

**DOI:** 10.17179/excli2017-978

**Published:** 2018-05-28

**Authors:** Junli Wang, Wenjing Xiao, Wanyi Chen, Yonghe Hu

**Affiliations:** 1 Department of Respiratory Medicine, 363 Hospital, Chengdu 610041, People's Republic of China; 2 Department of Pharmacy, Chengdu Military General Hospital, Chengdu 610083, People's Republic of China; 3 Chongqing Cancer Institute & Hospital & Cancer Center, Chongqing 400030, People's Republic of China

**Keywords:** glioma, neutrophil to lymphocyte ratio, platelet to lymphocyte ratio, prognosis

## Abstract

The neutrophil-to-lymphocyte ratio (NLR) and platelet-to-lymphocyte ratio (PLR) have been recognized as inflammatory markers and used as prognostic makers in various cancers. The present study sought to investigate the prognostic role of NLR and PLR in Chinese patients with glioma. Clinical data, including NLR, PLR and overall survival (OS), were collected from 112 patients who underwent surgery to treat primary glioma. Kaplan-Meier survival analysis as well as uni- and multivariate Cox regression were performed to examine potential associations of preoperative NLR and PLR with OS. Among all patients, mean NLR was 3.80±1.48 and mean PLR was 183.60±81.38. NLR increased with increasing WHO tumor grade (p < 0.05), but PLR did not (p > 0.05). Patients with NLR ≥ 4 had significantly shorter mean OS (20.75±7.68 months) than patients with NLR < 4 (26.91±7.50 months; p < 0.001). Similarly, patients with PLR ≥ LR had significantly shorter OS than patients with PLR < 200 (p = 0.007). Univariate Cox analysis identified the following parameters as significantly associated with worse OS: NLR (≥ 4), PLR (> 200), tumor size (≥ 5 cm), WHO grade (III/IV), and Karnofsky Performance Status (< 70). Multivariate analysis identified only NLR > 4 as an independent predictor of OS (HR 1.932, 95 % CI 1.011 to 3.694, p = 0.046). Our results suggest that at least in Chinese patients, increased preoperative NLR and PLR are associated with worse OS, and NLR may be an independent risk factor to identify glioma patients with poor prognosis. These results should be validated and extended in larger clinical studies.

## Introduction

Glioma is one of the most prevalent types of malignant brain tumor, and approximately 20,000 new gliomas were diagnosed in the US in 2015 alone (McNeill, 2016[11]). Glioma is characterized by high invasiveness and results in disappointing survival outcomes. Glioblastoma, in particular, is associated with median survival of one year and five-year mortality > 95 %, despite significant progress in neurosurgery, chemotherapy, and radiotherapy for treating and managing glioma (Zeng et al., 2015[20]; Bush et al., 2017[4]). The prognosis of Chinese patients with glioma is similarly poor (Yang et al., 2013[18]). Thus, simple and reliable prognostic markers are needed in order to predict clinical outcomes of patients with glioma and tailor their therapy accordingly. 

As in other malignant cancers, inflammation may contribute to glioma progression (Michelson et al., 2016[12]; Conti et al., 2010[5]). Biomarkers based on inflammation, such as the classical example of C-reactive protein, have been used to assess prognosis of glioma patients (Strojnik et al., 2014[14]). Neutrophil-to-lymphocyte ratio (NLR), a novel marker in many systemic inflammatory disorders (Faria et al., 2016[8]), reflects immune response arising from various stress stimuli. It has shown prognostic potential in several cancers, including lung, breast, and kidney cancer (Templeton et al., 2014[16]). Another inflammation biomarker, the platelet-to-lymphocyte ratio (PLR), has shown prognostic potential for patients with solid tumors (Templeton et al., 2014[15]). The present study sought to investigate whether pre-operative NLR and PLR are significantly associated with survival in Chinese patients with glioma. These results may help provide an evidence-based approach to stratifying patients by poor survival risk, thereby guiding the tailoring of treatment. 

## Patients and Methods

### Patients

A total of 112 patients with primary glioma who underwent surgery in our hospital between January 2010 and September 2013 were retrospectively enrolled in this study. Upon admission, all patients gave written informed consent that their medical records could be used for research purposes. A patient was included in this study if he or she (1) was diagnosed with glioma based on histopathology of surgical sections according to 2007 criteria from the World Health Organization (Louis et al., 2007[10]); (2) was subjected to routine blood analysis, including NLR and PLR, before surgery; (3) showed no signs of active infection, autoimmune, or hematological disorders; and (4) had not received steroid treatment before surgery. 

### Data collection

The following data were extracted from medical records: age at diagnosis, gender, tumor grade (I/II/III/IV by WHO classification), tumor size, and Karnofsky performance score (KPS). All patients underwent routine preoperative blood testing on an automated hematology analyzer (Abbott CD-1800; Abbott Laboratories, Abbott Park, IL, USA), which provided data on total white blood cell count, neutrophil count, platelet count, and lymphocyte count. NLR and PLR were calculated by dividing neutrophil or platelet count by lymphocyte count. Overall survival (OS) was defined as the interval from histopathology diagnosis until death or, in the case of surviving patients, until last follow-up in April 2016. 

### Statistical analysis

Continuous data are presented as mean ± standard deviation; categorical data, as frequencies and percentages. Inter-group differences were evaluated for significance using the nonparametric Mann-Whitney U test for continuous variables, and the chi-squared test for categorical variables. Differences among multiple groups were evaluated for significance using analysis of variance (ANOVA). Cutoff values of NLR and PLR for classifying patients as having "low" or "high" values were defined based on literature review and analysis of data in the present study. In the end, an NLR cutoff value of 4.00 and PLR cutoff value of 200.00 were used (Templeton et al., 2014[16][15]). Kaplan-Meier OS curves were plotted for patients stratified by low or high values of NLR and PLR. Survival differences between the two groups were evaluated for significance using the generalized log-rank test. Impact of NLR and PLR and other clinical variables on OS was evaluated using uni- and multivariate Cox regression. Hazard ratios (HR), together with 95 % confidence intervals (95 % CIs), were used to assess independent contributions of significant factors. All statistical analyses were performed using SPSS 18.0 (Chicago, IL, USA), with p < 0.05 considered significant.

## Results

### Clinical characteristics of patients

The entire study sample of 112 patients included 70 males and 42 females with mean age of 50±12 years (Table 1(Tab. 1)). A total of 43 patients (38 %) had tumors larger than 5 cm, and 47 (42 %) had KPS scores below 70. According to the WHO classification system, 59 patients (53 %) were classified as having low-grade (grade I-II) glioma, and 53 (47 %) as having high-grade (grade III-IV) glioma. Mean OS across all patients was 24.27±8.14 months. 

### NLR and PLR 

**Figure 1:**
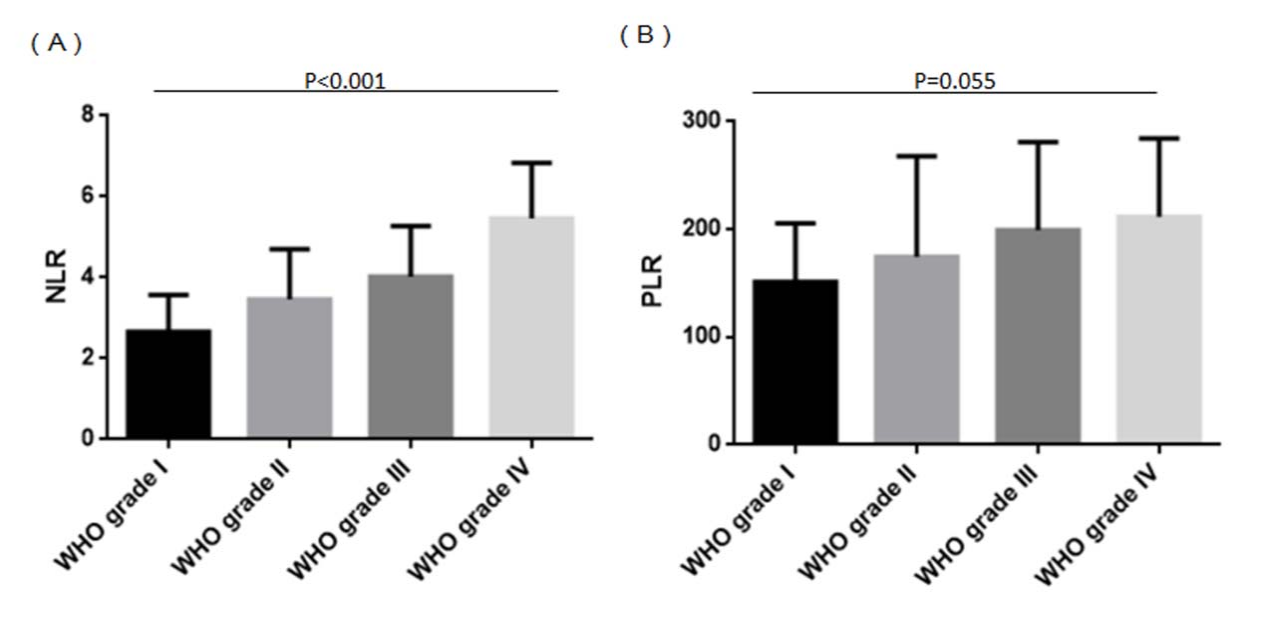
NLR and PLR in glioma patients stratified by WHO grade (A) NLR increased with increasing WHO grade (p < 0.001). (B) PLR showed a tendency to increase with WHO grade (p = 0.055). NLR: Neutrophil-to-lymphocyte ratio; PLR: Platelet-to-lymphocyte ratio; WHO: World Health Organization

Among all patients, mean NLR was 3.80±1.48 and mean PLR was 183.60. A total of 48 patients (43 %) had NLR > 4, and 36 (32 %) had PLR > 200. NLR levels increased significantly when moving from a lower to higher grade on the four-grade WHO scale (p < 0.001; Figure 1A(Fig. 1)). Similarly, PLR levels tended to increase with WHO grade, although this did not achieve statistical significance (p = 0.055; Figure 1B(Fig. 1)). Clinical characteristics of patients with high or low NLR and PLR are summarized in Table 1(Tab. 1).

### Kaplan-Meier OS analysis

**Figure 2:**
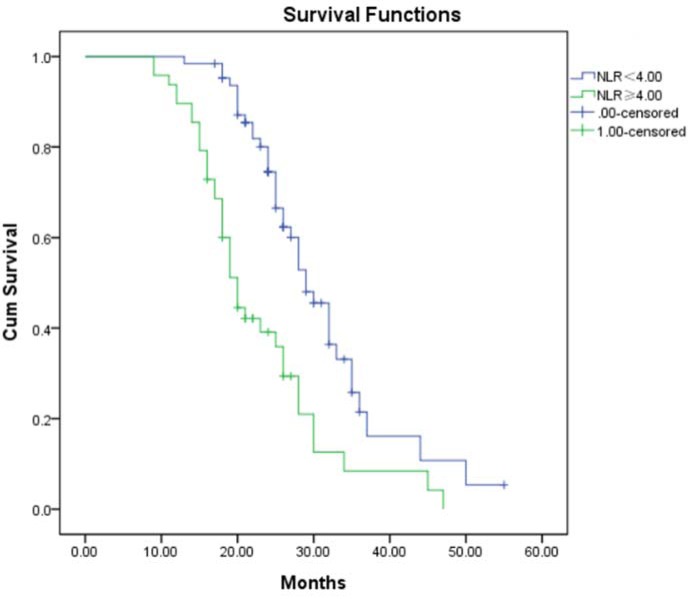
Kaplan-Meier analysis of overall survival of glioma patients stratified by NLR NLR: Neutrophil-to-lymphocyte ratio

**Figure 3:**
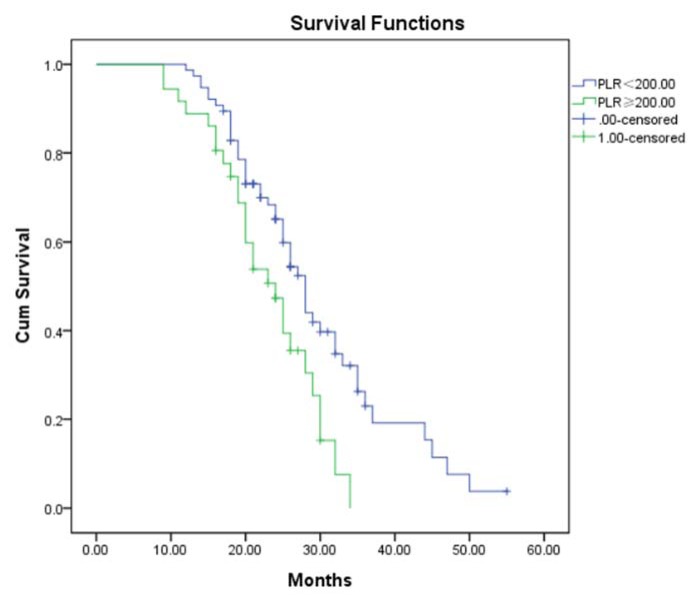
Kaplan-Meier analysis of overall survival of glioma patients stratified by PLR NLR: Neutrophil-to-lymphocyte ratio

Mean OS was significantly shorter for the 48 patients with NLR ≥ 4.00 (20.75±7.68 months) than for patients with NLR < 4.00 (26.91±6.91 months, p < 0.001; Figure 2(Fig. 2)). Mean OS was also significantly shorter for patients with high PLR ≥ 200 (21.61±6.35 months) than for those with PLR < 200 (25.53±8.62 months, p = 0.007; Figure 3(Fig. 3)).

### Cox regression 

The potential impact of factors on OS was assessed using Cox proportional hazard models. Univariate analysis identified the following as risk factors of poor OS (Table 2(Tab. 2)): tumor size ≥ 5 cm (HR 2.225, 95 % CI 1.399 to 3.539, p = 0.001), WHO grade III/IV (HR 1.637, 95 % CI 1.033 to 2.595, p = 0.036), KPS score < 70 (HR 2.247, 95 % CI 1.391 to 3.630, p = 0.001), NLR ≥ 4.00 (HR 2.577, 95 % CI 1.626 to 4.086, p < 0.001), and PLR ≥ 200 (HR 1.915, 95 % CI 1.171 to 3.132, p = 0.010). In contrast, multivariate Cox regression identified only NLR ≥ 4.00 as an independent prognostic factor for patients with glioma (HR 1.932, 95 % CI 1.011 to 3.694, p = 0.046).

## Discussion

Accurate prognostic evaluation of patients with glioma plays a critical role in their management, but it remains a clinical challenge. Here we provide evidence that, at least among Chinese patients with glioma, NLR ≥ 4.00 or PLR ≥ 200.00 may be associated with worse OS, and NLR may be an independent prognostic biomarker. This study is, to our knowledge, the first to assess the prognostic value of NLR and PLR in glioma in Chinese patients.

Abnormal inflammation is a characteristic of malignant cancers and a driver of malignant transformation of low-grade gliomas. Inflammation-induced activation of transcription factors contributes to the survival and rapid growth of glioma cells (Michelson et al., 2016[12]; Sen, 2011[13]). This systemic inflammation leads to a simultaneous increase in neutrophil count and decrease in lymphocyte count, which may reflect increased lymphocyte margination and apoptosis. The net result is inhibition of the cytolytic activity of immune cells and immunosuppression (el-Hag and Clark, 1987[7]). Increased NLR has been described as a novel inflammation biomarker with prognostic potential in several cancers (Templeton et al., 2014[16]). Consistent with this, NLR in our patients increased with WHO grade, suggesting that preoperative NLR may be useful for predicting glial tumor grade. Another study found that NLR correlates with glial brain tumor grade, and that NLR > 2.579 may be a discriminative parameter for predicting glioma grade (Zadora et al., 2015[19]). Our results show that Chinese patients with NLR ≥ 4.00 had significantly shorter OS based on Kaplan-Meier OS analysis, and multivariate Cox regression identified NLR as an independent prognostic factor in glioma. These results are similar to another study that found NLR > 4 to be an independent prognostic marker for poor outcome in patients with glioblastoma multiforme (Bambury et al., 2013[3]). However, our results contrast with a study in which multivariate Cox regression did not identify NLR as a prognostic biomarker in patients with glioma (Auezova et al., 2016[1]). More studies should be performed to validate our findings and assess the role of NLR in predicting the treatment response of patients with glioma, which would help guide their effective management and follow-up. 

Platelet-mediated inflammation contributes to cancer progression, so researchers have tested various platelet-based markers of inflammation in several cancers (Bambace and Holmes, 2011[2]). In glioblastoma patients, platelets induce angiogenesis of tumor endothelial cells and secretion of vascular endothelial growth factor, helping promote glioma progression. Platelet-derived growth factor contributes to oncogenic transformation and tumorigenesis (Di Vito et al., 2017[6]; Westermark et al., 1995[17]). Consistent with these studies on platelet-based indices in cancer, we found in the present study that Chinese glioma patients with PLR ≥ 200 had significantly shorter OS. However, Cox regression failed to identify PLR as an independent prognostic biomarker for glioma (HR 1.030, 95 % CI 0.559 to 1.896). This negative finding may reflect the influence of confounding factors, which should be explored in larger, preferably prospective studies aimed at clarifying the potential prognostic relevance of PLR in glioma.

Our data strongly suggest exploring NLR as a simple basis for assessing prognosis of patients with glioma. NLR can be calculated from inexpensive, routine blood analysis conducted on all patients. At the same time, studies should examine the reliability of NLR calculated from blood tests in the presence of potential interference from non-steroidal anti-inflammatory drugs, taken by patients with glioma to relieve headache (Imtiaz et al., 2012[9]). Future studies should also examine the possibility of monitoring NLR changes during glioma treatment in order to detect or predict early recurrence after surgery. 

There were several limitations in our study. First of all, we included only 112 patients, so our results require verification in larger studies. Second, we included only Chinese patients at a single center, so multi-site studies with patients of various ethnicities should be performed. Third, we did not have access to complete post-surgical treatment histories in this retrospective study, so other factors that we did not control may have contributed to the observed OS. To avoid these limitations, we suggest large, multi-site prospective studies to comprehensively evaluate the prognostic value of NLR and PLR in glioma. 

## Conclusion

This study found that among Chinese glioma patients, high NLR and PLR were associated with significantly worse OS, and that NLR may be an independent prognostic biomarker. Our study warrants larger trials involving multiple medical centers and patients of various ethnic groups in order to confirm the prognostic role of NLR in glioma.

## Notes

Junli Wang, Wenjing Xiao and Wanyi Chen contributed equally as first authors.

## Funding statement

This work was supported by the National Natural Science Foundation of China (Grant No. 81402507).

## Figures and Tables

**Table 1:**
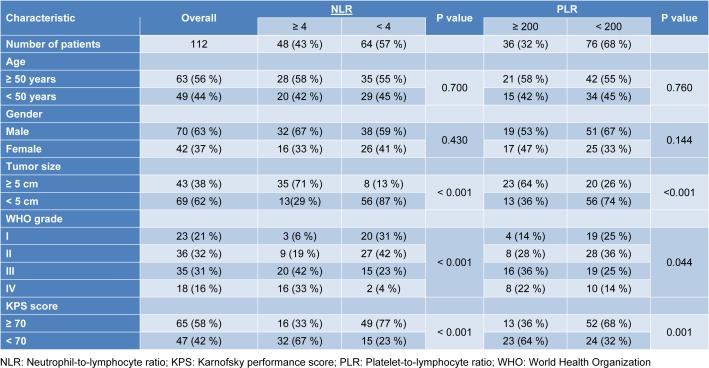
Clinical characteristics of Chinese glioma patients

**Table 2:**
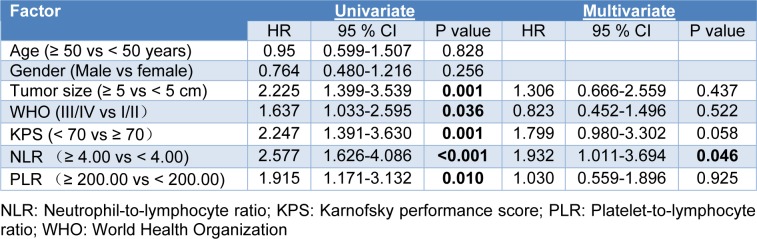
Uni- and multivariate Cox regression analysis of factors affecting overall survival
